# Supplementary material for: Electric fields control the orientation of peptides irreversibly immobilized on radical-functionalized surfaces
Source: Nat Commun. 2018 Jan 24;9:357. doi: 10.1038/s41467-017-02545-6 (PMC5783936; doi:10.1038/s41467-017-02545-6)
Supplement: Supplementary file 1 — Supplementary Information [file 41467_2017_2545_MOESM1_ESM.pdf]

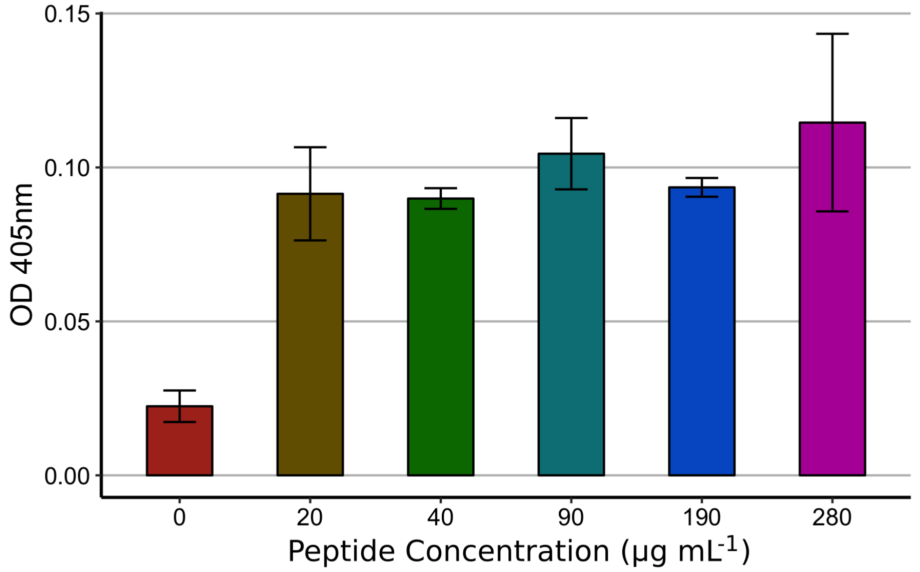

Supplementary Figure 1: Absorbance saturates at high peptide concentration. Enzyme-linked immunosorbent assay does not show concentration dependence above 20 mg mL^{-1}, indicating the signal is already saturated. Concentrations above 20 μg mL^{-1} are rounded to the nearest 10 μg mL^{-1}. Error bars are s.d.

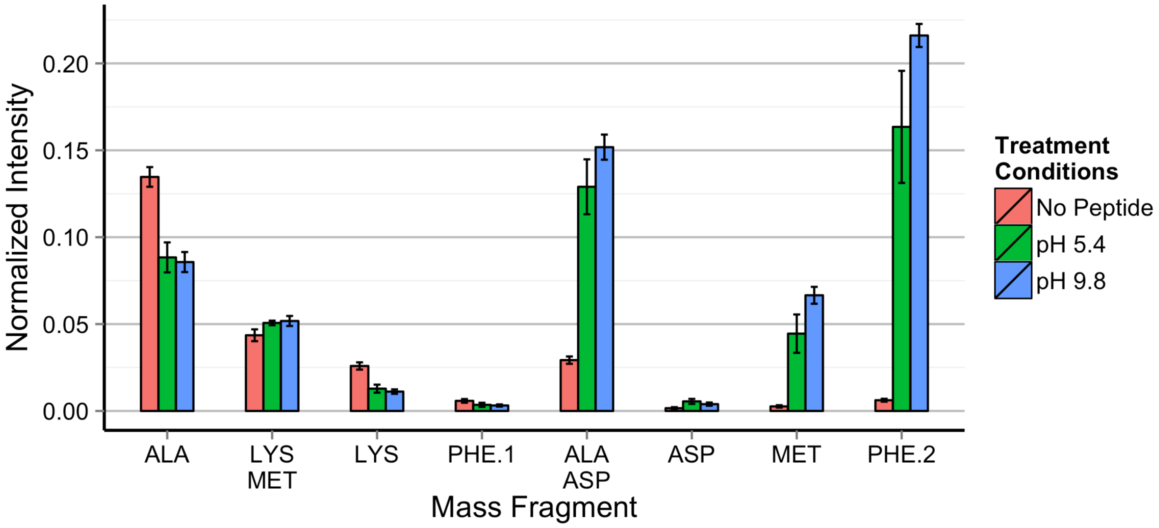

Supplementary Figure 2: Mass spectroscopy of the surface peptide. Time of flight secondary ion mass spectroscopy mass fragments of all residues from the FLAG peptide. The first four fragments (ALA, LYS/MET, LYS, and PHE.1) are discarded because they are present in the No Peptide case. This is because they arise from the radical-functionalized plasma polymer surface itself, confusing the analysis of the pH 5.4 and pH 9.8 cases. The ALA/ASP and ASP fragments are also discarded because the differences in intensity cannot be unambiguously attributed to either ALA or ASP, since they have an unknown contribution to the ALA/ASP fragment. The remaining fragments, MET and PHE.2, are used to analyse peptide orientation. Error bars are s.d.
